# The Seizure‐Associated Genes Across Species (SAGAS) database offers insights into epilepsy genes, pathways and treatments

**DOI:** 10.1111/epi.17352

**Published:** 2022-07-10

**Authors:** Lara Gracie, Danial Rostami‐Hochaghan, Basel Taweel, Nasir Mirza, the SAGAS Scientists' Collaborative, Abdal‐Aziz Agbabiaka, Ayra Ahmed, Tamara Al‐Bahri, Michelle Alexander, Lucky Ali, James Ashton, Zahra Aslam, Alexander Benson, Mahima Bharadwaj, George Robert Biggin, Bianca Bileca, Fiona Bradley, Thomas Carpenter, Tabitha Champion, Mark Chisnall, Megan Davies, Alan Mathew George, Jayden Anthony Gittens, Benjamin Goriely‐McDonald, Kartik Goyal, Lauren Hall, Yousif Hufthy, Rebecca Humphreys, Nafisa Hussain, Ovin Baratha Jayawardena, Hayley Jones, Madhia Khaliq, Alizah Khan, Daanish Khan, Zara Khan, Claudia Kirby, Thea Louise Emilia Jones, Amaya Malmalabaduge, Leia Martin, Sousan Mayou, Mohammad Mustafa, Arman Nabavieh, Sayeda Tajmun Nahar, Sahana Narayan, Deimante Narusyte, Elena Panayi, Thomas Posner, William Thomas Purcell, Emily Raw, Megan Roberts, Menahel Sukkena Sajjad, Abdul‐Wahab Shaheen, Areej Shams‐Khan, Callum Swift, Alistair Topping, David Uchegbu, Maddalena Ventre, Alexander Walmsley, Chloe Williams, Thomas Woodruff, Nicole Yap

**Affiliations:** ^1^ Birmingham Medical School University of Birmingham Birmingham UK; ^2^ School of Medicine University of Liverpool Liverpool UK; ^3^ Department of Pharmacology & Therapeutics, Institute of Systems, Molecular and Integrative Biology University of Liverpool Liverpool UK

**Keywords:** antiepileptic drug, antiseizure drug, database, drug repurposing, epilepsy, gene, pathway, protein, seizure

## Abstract

**Objective:**

Decades of genetic studies on people with many different epilepsies, and on many nonhuman species, using many different technologies, have generated a huge body of literature about the genes associated with seizures/epilepsy. Collating these data can help uncover epilepsy genes, pathways, and treatments that would otherwise be overlooked. We aimed to collate and structure these data into a database, and use the database to identify novel epilepsy genes and pathways, and to prioritize promising treatments.

**Methods:**

We collated all the genes associated with all types of seizures/epilepsy in all species, and quantified the supporting evidence for each gene, by manually screening ~10 000 publications, and by extracting data from existing databases.

**Results:**

The largest published dataset of epilepsy genes includes only 977 genes, whereas our database (www.sagas.ac) includes 2876 genes, which demonstrates that the number of genes that can potentially contribute to seizures/epilepsy is much higher than previously envisaged. We use our database to identify 12 hitherto unreported polygenic epilepsy genes, 479 high‐confidence monogenic epilepsy genes, and 394 more biological pathways than identified using the previously largest epilepsy gene dataset. We use a unique feature of Seizure‐Associated Genes Across Species—the number of citations for each gene—to demonstrate that a drug is more likely to affect seizures if there is more evidence that the genes it affects are associated with seizures, and we use these data to identify promising candidate antiseizure drugs.

**Significance:**

This database offers insights into the causes of epilepsy and its treatments, and can accelerate future epilepsy research.

**Key Points**

- We collated all the genes associated with all types of seizures or epilepsies in all species, and quantified the supporting evidence for each gene
- The largest published dataset of epilepsy genes includes only 977 genes, whereas our database (www.sagas.ac) includes 2876 genes
- We use our database to identify genes and pathways associated with monogenic and polygenic epilepsies, and candidate antiseizure drugs
- This database offers insights into the causes of epilepsy and its treatments, and can accelerate future epilepsy research

## 1 INTRODUCTION

Seizures are the hallmark of epilepsy, a condition that affects almost 1% of the global population, and places a heavy burden on individuals and societies.^1^ Better understanding of the genes and genetic pathways underlying epilepsy can help improve the diagnosis and management of people with epilepsy. Genetic factors can contribute to the development of epilepsies, either as single‐gene mutations in rare monogenic epilepsies, or as multiple genetic variants in common epilepsies.^2^ Common epilepsies are complex traits with a polygenic origin, which means that the combined effect of many common risk variants contributes to their genetic risk.^2^ Seizures or epilepsy can also be observed or elicited in other animals, including nonhuman primates, dogs, cats, rodents, fish, flies, and worms.

Seizure or epilepsy genes have been identified through studies on people with polygenic epilepsies, and on people with monogenic epilepsies, and on the many nonhuman species that can also experience seizures or epilepsy. Decades of genetic research using numerous different technologies in multiple species of animals with many different types of epilepsy have generated a huge body of literature about the genes associated with seizures and epilepsy. It is evident from this literature that the dysfunction of a multitude of different genes can provoke or predispose to seizures and/or epilepsy. Some genes can influence seizure susceptibility in different types of epilepsy and across species. The genes that can cause epilepsy of different types and/or in different species are, putatively, particularly potent epilepsy‐causing genes and, hence, deserving of particular interest.

Existing epilepsy gene databases are limited to genes associated with monogenic human epilepsies. Data for the strength of genes' association with polygenic types of epilepsy are overlooked. Data from studies in nonhuman animal models, which also provide vital insights into the genes/proteins that influence seizure susceptibility, are not included. No data are provided to rank genes, which would help prioritize the most promising candidate genes, given the vast number of genes associated with epilepsy.

Genetic findings from different epilepsy types, animal species, and analytical techniques are deposited in disparate databases or dispersed across a multitude of papers published over many decades. The vast magnitude and dispersed nature of this literature means that individual genes that are important leads for further study get overlooked, the set of genes with the greatest evidence of association with seizures and epilepsy cannot be identified, and a global view of the genetic pathways driving epilepsy cannot be formulated. Collating, summarizing, categorizing, enumerating, integrating, and juxtaposing published information for different types of epilepsy and animal species into a single database can address these needs and, thereby, help to accelerate mechanistic and therapeutic discovery in epilepsy. Our aim was to create such a database of all the genes associated with all types of seizures or epilepsies in all species.

## 2 MATERIALS AND METHODS

We searched Scopus for studies reporting one or more named genes whose mutation or manipulation led to seizures/epilepsy in people or animal models. We included studies reporting an association between seizures/epilepsy and mutation(s), variation(s), or polymorphism(s) in one or more named genes. In addition, we included articles demonstrating that direct manipulation (for example, gene knockout, RNA interference, Targeted Augmentation of Nuclear Gene Output) of one or more named genes/proteins caused seizures or epilepsy. Administration of drugs or pharmacological compounds that affect a protein/gene were not accepted, as compounds can also influence proteins/genes other than the target gene and, therefore, it cannot be definitively concluded that the effect of the compound on the intended target is the sole cause of the observed phenotype in studied subjects. Studies reporting clinical or electrical seizures were eligible.

Separate searches were conducted for human and for animal model studies. For animal studies, we included all years to December 15, 2020. For human studies, we included all years from January 1, 2016 to December 15, 2020. Earlier years were not included in the search for human studies, as human studies up to and including 2015 had been collated by two previously published comprehensive human epilepsy gene databases/datasets, whose data was downloaded/extracted in bulk (see below).

The Scopus search yielded 9541 articles, which were then manually screened and mined. Given the large number of articles, crowdsourcing was employed. Sixty researchers contributed to the crowdsourced manual screening and data mining. The researchers were provided instructions and training to perform the task, and the quality of their work was audited by the senior team. To standardize the article screening and data extraction process, an online systematic review platform (https://sysrev.com/) was used. Written guidelines were created to standardize study selection and data extraction by all collaborators. A copy of the guidelines can be downloaded from the following link: https://figshare.com/s/6394da0063971fb5a3bc. Each article was screened independently by at least two researchers. Any conflicts were resolved by a team of four senior researchers. Finally, all collated information was tabulated and rechecked for errors.

Data were also extracted (on May 26, 2021) from the following databases: ClinVar (https://www.ncbi.nlm.nih.gov/clinvar/), Online Mendelian Inheritance in Man (https://www.omim.org/), Human Gene Mutation Database (https://digitalinsights.qiagen.com/products‐overview/clinical‐insights‐portfolio/human‐gene‐mutation‐database/), Mouse Genome Informatics (http://www.informatics.jax.org/), International Mouse Phenotyping Consortium (https://www.mousephenotype.org/), EpilepsyGene (http://www.wzgenomics.cn/EpilepsyGene/),^3^ and epiGAD (http://www.epigad.org/). Data from a comprehensive review of epilepsy‐associated genes were also manually extracted.^4^ Any nonhuman genes were mapped to the orthologous human genes.

Genome‐wide association study (GWAS) gene‐based *p*‐values were calculated for the two main types of common epilepsy—focal and generalized—from their GWAS summary statistics^5^ using FUMA (https://fuma.ctglab.nl/) with default settings.

It was deemed important that the database's online interface should fulfill the following requirements:

- The database should be presented like a spreadsheet, because this best enables users to take advantage of a useful feature of the database—numerical quantification of the evidence/citations for the association of each gene with epilepsy/seizures in different species—to sort, prioritize, and select the most promising genes.
- The database should include an advanced filtering tool, so that it is possible to identify genes that have the required amount of evidence, as defined by the user, in any or all of different epilepsy types and animal species.
- The filtered results should be downloadable by the user.
- The citations reporting an association between seizures/epilepsy and any individual gene should be immediately easily viewable.
- The database should be browsable using a mobile device (with a large screen).

A number of proprietary platforms for hosting spreadsheetlike databases were explored, but none met all of the above requirements. Hence, the database was created in Excel, and customized using the Visual Basic for Applications programming language, to meet the above requirements.

Next, we used the database to identify epilepsy genes, pathways, and treatments, to deliver new mechanistic and therapeutic insights, and to provide examples of the database's utility.

### 2.1 Revealing genes associated with polygenic epilepsies

We identified genes that have a GWAS gene‐based false discovery rate (FDR) < .05 and evidence of association with epilepsy/seizures in monogenic cases/models. Gene ontologies for the identified genes were extracted from http://geneontology.org/ (accessed October 10, 2021).

### 2.2 Identifying "high‐confidence" monogenic human epilepsy genes

High‐confidence monogenic human epilepsy genes were defined as those with evidence of association with monogenic epilepsy in humans and with seizures/epilepsy in another species.

### 2.3 Revealing the pathways underlying seizures/epilepsy

Pathway analysis was performed using all of the genes in Seizure‐Associated Genes Across Species (SAGAS) and the hypeR Bioconductor package and Reactome pathways.^6^ To determine whether SAGAS identifies more epilepsy pathways than can be identified by chance or by previous databases, pathway enrichment analysis was also done using random genes equal in number to those in SAGAS (10^3^ permutations performed), and using all of the genes in the previously largest epilepsy gene dataset.^4^

### 2.4 Identifying potential antiseizure medications

We hypothesized that a drug is more likely to affect seizures if there is more evidence that the genes it affects are associated with seizures. We ranked drugs by the total number of citations in SAGAS for all the genes that each drug affects, and determined whether this leads to a statistically significantly enrichment of antiseizure drugs.

We created a list of compounds that are promising potential antiseizure drugs, as they (1) are already used to treat people (for conditions other than epilepsy), (2) affect epilepsy genes, and (3) have evidence of efficacy against seizures in animal models.

Genes/proteins affected by drugs were collated from publicly accessible databases, as previously described.^7^ The list of currently approved antiseizure drugs was extracted from the British National Formulary (https://bnf.nice.org.uk/treatment‐summary/epilepsy.html; accessed October 10, 2021). The list of experimental antiseizure drugs, and the supporting evidence, were extracted from a published database.^8^

## 3 RESULTS

Our database contains 2876 genes, supported by 9742 pieces of evidence. Our database is available at (www.sagas.ac).

### 3.1 Database interface

In the database, genes can be sorted/filtered by degree of association with either of the two main types of polygenic epilepsy (focal and generalized) and/or the total number of studies reporting an association with monogenic epilepsies across all species or in specific species (human, dog, rodent, zebrafish, fly, worm). Where available, the seizure or epilepsy type or syndrome associated with the gene is displayed. Searching by gene or syndrome name is also possible. For any gene of interest, a single click displays the list of citations reporting its association with seizures/epilepsy. Each citation can be clicked to directly open the relevant PubMed page or database.

### 3.2 Revealing genes associated with polygenic epilepsies

Using the comprehensive collection of data in SAGAS, we identified 16 genes that are significantly associated with generalized epilepsy (GWAS gene‐based FDR < .05) and cause epilepsy/seizures in monogenic cases/models and, hence, are promising candidate causal generalized epilepsy genes (Table 1). Twelve of these genes were not reported in the original GWAS paper and, hence, are important newly reported generalized epilepsy candidate genes: *AP3D1, CAMTA1, DOC2A, GRM4, MMP27, PHACTR1, RAPGEF2, RBFOX1, RIMS1, SETD1A, STX1B, UBTF*.

**TABLE 1:** Genes that are significantly associated with generalized epilepsy and cause epilepsy/seizures in monogenic cases/models and, hence, are promising candidate causal generalized epilepsy genes

Gene	Citations, *n*	GE FDR	GO terms (selected)
*AP3D1*	5	.001574	Anterograde synaptic vesicle transport
*CAMTA1*	2	.002134	Calcineurin‐NFAT signaling
*SCN1A*	290	.003081	Voltage‐gated sodium channel activity
*SETD1A*	3	.011449	Protein binding
*STX1B*	8	.021646	Signaling receptor binding
*RAPGEF2*	4	.028498	Guanyl‐nucleotide exchange factor activity
*UBTF*	2	.031009	RNA polymerase I cis‐regulatory region sequence‐specific DNA binding
*GABRA2*	9	.032305	GABA‐gated chloride ion channel activity
*PCDH7*	1	.033095	Calcium ion binding
*RIMS1*	1	.033095	Synaptic vesicle exocytosis
*PHACTR1*	2	.03322	Actin cytoskeleton organization
*RBFOX1*	5	.037034	Protein binding
*TTC21B*	2	.038402	Positive regulation of canonical Wnt signaling pathway
*MMP27*	1	.043774	Zinc ion binding
*DOC2A*	1	.047941	Calcium ion binding and syntaxin binding
*GRM4*	4	.049296	G protein‐coupled glutamate receptor signaling

*Note*: Number of citations is from Seizure‐Associated Genes Across Species database. Abbreviations: GABA, γ‐aminobutyric acid; GE FDR, false discovery rate for association with generalized epilepsy (see text); GO, gene ontology; NFAT, nuclear factor of activated T cells.

In the focal epilepsy GWAS, no genes were significantly associated (gene‐based FDR < .05). No significant associations with focal epilepsy were discovered by the original GWAS publication either.^5^

### 3.3 Identifying high‐confidence monogenic human epilepsy genes

A total of 479 high‐confidence monogenic human epilepsy genes, as defined above, were identified (Table S1).

### 3.4 Revealing the pathways underlying seizures/epilepsy

Pathway enrichment analysis using all of the genes in the previously largest epilepsy gene dataset^4^ revealed 267 significantly enriched pathways. Pathway enrichment analysis using all of the genes in SAGAS revealed 661 significantly enriched pathways (Tables 2 and S3). Pathway enrichment analysis using random genes equal in number to those in SAGAS did not reveal as many significantly enriched pathways; we repeated this analysis 10^3^ times, giving *p* < 1 × 10^3^ for as many significantly enriched pathways being revealed by chance.

**TABLE 2:** Selected enriched biology pathways identified using all of the genes in the SAGAS database

Pathway	FDR
L1CAM interactions	7.8 × 10^−26^
Asparagine N‐linked glycosylation	4.0 × 10^−17^
NCAM signaling for neurite out growth	1.1 × 10^−14^
Signaling by NTRKS	1.1 × 10^−14^
CREB1 phosphorylation through NMDA receptor‐mediated activation of RAS signaling	8.0 × 10^−14^
Interaction between L1 and ankyrins	1.5 × 10^−13^
MAPK family signaling cascades	2.5 × 10^−12^
Citric acid TCA cycle and respiratory electron transport	3.0 × 10^−12^
G alpha (i) signaling events	3.0 × 10^−11^
Signaling by VEGF	3.2 × 10^−11^
RAS activation upon CA2 influx through NMDA receptor	2.5 × 10^−10^
Synthesis of glycosylphosphatidylinositol	3.6 × 10^−10^
PI3K AKT signaling in cancer	3.9 × 10^−10^
Signaling by PDGF	5.5 × 10^−10^
Regulation of MECP2 expression and activity	7.9 × 10^−10^

*Note*: The complete list of enriched biological pathways can be found in Tables S1–S5. Abbreviations: FDR, false discovery rate; SAGAS, Seizure‐Associated Genes Across Species.

### 3.5 Identifying potential antiseizure medications

To test the hypothesis that that a drug is more likely to affect seizures if there is more evidence that the genes it affects are associated with seizures, we ranked drugs by the total number of citations in SAGAS for all the genes that each drug affects. Ranking drugs in this way places 100% of antiseizure drugs within the top 20% of all drugs, which is fivefold and highly statistically significant (hypergeometric *p*‐value = 6.1 × 10^−20^) enrichment. In Tables 3 and S4, we list drugs that are particularly promising potential antiseizure drugs as they (1) are already used to treat people (for conditions other than epilepsy), (2) affect epilepsy genes, and (3) have evidence of efficacy against seizures in animal models.

**TABLE 3:** Selected drugs that affect genes in the SAGAS database and have evidence of antiseizure efficacy in multiple animal models and from multiple published studies

Drug	Current indications (selected)	Citations	Studies	Models
Riluzole	Amyotrophic lateral sclerosis	482	4	4
Memantine	Alzheimer disease	322	19	5
Verapamil	Angina, hypertension, cluster headache	165	12	6
Meperidine	Pain	155	3	2
Amlodipine	Hypertension, angina	152	4	3
Dextromethorphan	Inflammatory and allergic disorders, congenital adrenal hyperplasia, croup, rheumatic disease	145	22	6
Nifedipine	Angina, hypertension, Raynaud phenomenon, premature labor	86	22	6
Fluoxetine	Depression, bulimia nervosa, obsessive–compulsive disorder	54	11	3
Scopolamine	Motion sickness, excessive respiratory secretions	54	8	3
Aripiprazole	Schizophrenia	52	3	3
Bromocriptine	Parkinson disease, endocrine disorders	52	3	2
Pimozide	Schizophrenia	51	3	4
Apomorphine	Parkinson disease	42	10	4
Bumetanide	Edema	28	5	4
Celecoxib	Osteoarthritis, rheumatoid arthritis, ankylosing spondylitis	25	4	2
Dexamethasone	Inflammatory and allergic disorders, congenital adrenal hyperplasia, cerebral edema	25	3	3

*Note*: "Citations" is the total citations in SAGAS for genes affected by the drug; "Studies" is the number of studies demonstrating antiseizure efficacy of the drug in animal models; "Models" is the number of unique animal models in which the drug has antiseizure efficacy. The complete list of drugs, and PubMed identifiers for the animal model studies reporting their antiseizure efficacy, can be found in Table S4. Abbreviation: SAGAS, Seizure‐Associated Genes Across Species.

## 4 DISCUSSION

Databases/datasets of epilepsy genes have been published previously; these are described in Table S5. The following unique features make our database different from and substantially better than previously published databases/datasets of epilepsy genes:

- The largest published dataset of epilepsy genes includes only 977 genes, whereas our database includes 2876 genes. Our database demonstrates that the number of genes that can potentially contribute to seizures/epilepsy is much higher than previously envisaged.
- Previously published epilepsy databases/datasets are limited to genes that cause monogenic forms of human epilepsy. Our database also incorporates the strength of association of each gene with polygenic forms of human epilepsy.
- Previously published epilepsy databases/datasets present only data from gene mutation/variation analyses in people with epilepsy. The discovery of a gene mutation in an individual with epilepsy is not always sufficient to establish that the gene causes epilepsy. For all genes found to bear mutations/variations in people with epilepsy, we also present any existing evidence of their association with seizures in animal models, which can be critical for establishing the genes' pathogenicity.
- We also include genes that have evidence of association with seizures/epilepsy in animals, but do not yet have such evidence in humans. These genes are potentially important leads for mechanistic, diagnostic, and therapeutic discovery in human epilepsy.
- Previously published epilepsy databases/datasets are limited to studies of inherited or de novo gene mutations/variations. We have also included relevant data from gene manipulation studies that used techniques such as gene knockin, RNA interference, and CRISPR (clustered regularly interspaced short palindromic repeats).
- For each gene, our database displays a numeric value for the strength of its association with polygenic forms of epilepsy, and the number of articles of evidence that show its association with monogenic seizures/epilepsy in each animal species. This allows users to rank genes according to the amount of evidence of their association with seizures/epilepsy, and to select the genes that meet any threshold chosen by the user.
- It is possible to identify genes that have evidence of association with seizures/epilepsy in one or more specific species of interest and that have the desired number of articles of evidence for the species.

### 4.1 Revealing genes associated with polygenic epilepsies

GWAS is an important methodology for identifying genes associated with polygenic forms of a disease. In GWAS, genetic variants/polymorphisms associated with the disease are identified and then mapped to genes. However, even some associations that meet stringent criteria for statistical significance will be spurious. If, through GWAS, a gene is found to be significantly associated with the polygenic form of a disease, and it is also known to cause the disease in monogenic cases/models, its association with the polygenic form of the disease is more likely to be true. SAGAS allows rapid identification of genes that are associated with polygenic epilepsies and that also cause epilepsy/seizures in monogenic cases/models. Using SAGAS in this way, we identified a number of generalized epilepsy genes, whose association with generalized epilepsy has not been reported previously. These include *AP3D1*, which is involved in anterograde synaptic vesicle transport, *GRM4*, which is involved in G protein‐coupled glutamate receptor signaling, and *RIMS1*, which is involved in synaptic vesicle exocytosis.^9^ SAGAS will be updated once the results of the next epilepsy GWAS become available.

### 4.2 Identifying "high‐confidence" monogenic human epilepsy genes

Not all mutations identified in individuals with monogenic or Mendelian diseases are pathogenic; some are spurious findings. Hence, it is desirable to identify high‐confidence genes that are more likely to be truly pathogenic. In a previous epilepsy database,^3^ the authors defined high‐confidence genes using the following method: different bioinformatics tools were used to score the likelihood that a gene mutation/variation will alter the resulting protein, then these scores were summed, and then an arbitrary cutoff was applied to the total score. A total of 154 high‐confidence epilepsy genes were thus identified. We exploit the wealth of data in SAGAS to identify high‐confidence genes using functional evidence; we consider a gene that has evidence of association with human monogenic epilepsy to be a high‐confidence gene if there is functional evidence that mutation/manipulation of the gene also causes seizures/epilepsy in another species. Arguably, these functional data are more robust evidence of pathogenicity than the computational predictions previously employed. Moreover, we identify a higher number of high‐confidence genes: 479 (Table S1). At the same time, we acknowledge that there is currently no single universally accepted method for identifying high‐confidence genes. Mutations in some genes might be pathogenic in humans only, or might not have been studied in animals, and such genes will not be included in our list of high‐confidence genes. Genes that are identified as high‐confidence using contrasting/complementary methods will be of particular interest. Hence, in Table S2, we present genes that are identified as high‐confidence by both the computational predictions in the previous database^3^ and the functional data in our database. For comparison, Table S2 also lists the genes identified as high‐confidence by only one of the databases. A Venn diagram summarizing the number of overlapping and unique high‐confidence genes in the two databases is displayed alongside Table S2.

Importantly, SAGAS provides the data and capability for users to apply alternative custom definitions of high‐confidence genes that are more suited to their research goals.

### 4.3 Revealing the pathways underlying seizures/epilepsy

SAGAS provides novel insights into the biological pathways underlying seizures/epilepsy. The breadth of insight into the biological pathways underlying seizures/epilepsy that is provided by SAGAS cannot be provided by previous epilepsy gene databases; SAGAS enables the identification of ~2.5‐fold more enriched pathways than the previously largest epilepsy gene database. The large number of enriched pathways identified using SAGAS is not expected by chance (*p* < 1 × 10^3^ by permutation).

Among the enriched pathways are pathways responsible for the expression/activity of genes that are known to be associated with epilepsy. For example, one of the enriched pathways is "regulation of *MECP2* expression and activity." Mutations in *MECP2* cause not only Rett syndrome but also numerous other syndromes in which epilepsy and seizures are a prominent feature (please see the SAGAS database for a complete list of the syndromes and related references). Another example is the pathway "*L1CAM* interactions." *L1CAM* mutations cause L1 syndrome, in which seizures are a prominent feature (please see the SAGAS database for related references).

Also among the enriched genetic pathways are pathways responsible for the expression/activity of genes that are not known to be associated with epilepsy. For example, mutations and variations in *NCAM* have not been associated with epilepsy. However, *NCAM* is involved in neural differentiation and synaptic plasticity.^9^ The "*NCAM* signaling for neurite out growth" pathway is one of the most highly enriched pathways in our analysis, as >50% of the genes in this pathway are found in our database (Table S3). As another example, mutations and variations in *VEGF* have not been associated with epilepsy. *VEGF* exerts neurotrophic and neuroprotective effects in the nervous system.^10^ The "*VEGF* signaling" pathway is one of the most highly enriched pathways in our analysis, as ≈40% of the genes in this pathway are found in our database (Table S3).

### 4.4 Identifying potential antiseizure medications

We used a unique feature of SAGAS—the number of citations for each gene—to prioritize drugs that affect seizures. We found that a drug is more likely to affect seizures if there is more evidence that the genes it affects are associated with seizures. One possible explanation of this finding is that, to treat epilepsy, drugs have been developed that specifically target genes associated with epilepsy. Even if this explanation is true, it suggests that identifying drugs that target epilepsy‐associated genes has been a fruitful strategy for finding antiseizure drugs in the past and, hence, should be a fruitful strategy for finding antiseizure drugs in the future.

Current drug treatments for epilepsy fail to control seizures in ~30% of patients^11^, ^12^ and cause adverse effects in ~88% of patients.^13^, ^14^ Hence, there is an urgent need for new antiseizure drugs. Developing and obtaining regulatory approval for a new drug takes 15 years and costs $2.6 billion. Repurposing drugs that are already being used to treat people (for conditions other than epilepsy) can meet this urgent need much more rapidly and economically. In Tables 3 and S4, we list compounds that are particularly promising candidates for repurposing as antiseizure drugs because they (1) are already used to treat people (for conditions other than epilepsy), (2) affect epilepsy genes, and (3) have evidence of efficacy against seizures in animal models. Some of these drugs even have published evidence of efficacy in case series of people with epilepsy, for example, memantine,^15^, ^16^, ^17^, ^18^ nifedine,^19^, ^20^, ^21^, ^22^, ^23^, ^24^ riluzole,^25^ and verapamil.^26^, ^27^, ^28^, ^29^, ^30^, ^31^, ^32^ Of course, these drugs have to be tested and proven effective in people with epilepsy, through future clinical trials, before being routinely deployed in the clinic.

In Table S4, we display the total number of citations in SAGAS for the genes affected by each drug, the number of studies reporting the efficacy of each drug in animal models of seizures, and the number of different animal models of seizures in which each drug is effective. Users can use these criteria to prioritize and select promising potential antiseizure drugs.

Limitations

Despite our best efforts, it is possible that some genes and/or studies that should be included in the database have been omitted, and that some genes and/or studies that do not belong in the database have been included. We have not assessed the quality of the studies included in the database, and it is possible that some of the reported gene associations with seizures/epilepsy are spurious. However, direct links to the evidence are provided in the database, so that users can easily access and review the evidence for any gene of interest. Our database is not intended to replace expert‐selected sets of the key genes that are most likely to cause human epilepsy (e.g., https://panelapp.genomicsengland.co.uk/panels/402/).

To ensure that our collection of seizure/epilepsy genes is as complete as possible, we have extracted data from and cited primary publications and gene databases. It is possible that the citation count for a gene is overestimated, because we have cited both the primary publication reporting its association with seizures/epilepsy and a database that has included the gene based on the same primary publication. However, as the primary purpose of the citation count is to identify genes that are more likely to be associated with seizures/epilepsy, even such a duplicated citation count can be useful, as it indicates that other researchers have also adjudged that a primary publication evidences the genes' association with seizures/epilepsy. A higher citation count can indicate that a gene is more likely to be truly associated with seizures/epilepsy, but the citation counts are not directly proportional to this likelihood.

Genes that cause seizures in a nonhuman species might not cause seizures in humans.

## 5 CONCLUSIONS

We have created the largest ever seizures/epilepsy gene database, and the only one that includes genes associated with different types of seizures/epilepsy in multiple species based upon a wide range of experimental techniques. The database provides novel insights into epilepsy genes, pathways, and treatments. The database can accelerate future mechanistic and diagnostic discoveries in epilepsy. We plan to update the database biennially. This database can also be used as a blueprint to create similar databases for other diseases.

## AUTHOR CONTRIBUTIONS

N.M. conceptualized and designed the project. All authors collected data, and reviewed and approved the manuscript. L.G. and D.R.‐H. led the crowdsourced data mining. B.T. created the database from the collated data. L.G., D.R.‐H., and B.T. checked the database for errors. B.T. and N.M. performed the bioinformatics analyses, and wrote the first draft of the paper. All authors approved the final draft of the manuscript.

## CONFLICT OF INTEREST

The authors declare that they have no competing interests. We confirm that we have read the Journal's position on issues involved in ethical publication and affirm that this report is consistent with those guidelines.

## Supporting information


TABLE S1
